# In Vitro and In Silico Based Approaches to Identify Potential Novel Bacteriocins from the Athlete Gut Microbiome of an Elite Athlete Cohort

**DOI:** 10.3390/microorganisms10040701

**Published:** 2022-03-24

**Authors:** Laura Wosinska, Calum J. Walsh, Paula M. O’Connor, Elaine M. Lawton, Paul D. Cotter, Caitriona M. Guinane, Orla O’Sullivan

**Affiliations:** 1 Department of Biological Sciences, Munster Technological University, Cork Campus, T12 P928 Cork, Ireland; laura.wosinska@teagasc.ie (L.W.); caitriona.guinane@mtu.ie (C.M.G.); 2 Teagasc Food Research Centre, Moorepark, Fermoy, P61 C996 Cork, Ireland; calum.walsh@unimelb.edu.au (C.J.W.); paula.oconnor@teagasc.ie (P.M.O.); elaine.lawton@mtu.ie (E.M.L.); paul.cotter@teagasc.ie (P.D.C.); 3 APC Microbiome Ireland, T12 YT20 Cork, Ireland; 4 VistaMilk, Fermoy, P61 C996 Cork, Ireland

**Keywords:** bacteriocins, microbiome, antimicrobial-peptides, athletes, in vitro, in silico, metagenomics

## Abstract

Exercise reduces inflammation, fatigue, and aids overall health. Additionally, physical fitness has been associated with desirable changes in the community composition of the athlete gut microbiome, with health-associated taxa being shown to be increased in active individuals. Here, using a combination of in silico and in vitro methods, we investigate the antimicrobial activity of the athlete gut microbiome. In vitro approaches resulted in the generation of 284 gut isolates with inhibitory activity against *Clostridioides difficile* and/or *Fusobacterium nucleatum,* and the most potent isolates were further characterized, and potential bacteriocins were predicted using both MALDI-TOF MS and whole-genome sequencing. Additionally, metagenomic reads from the faecal samples were used to recover 770 Metagenome Assembled Genomes (MAGs), of which 148 were assigned to be high-quality MAGs and screened for the presence of putative bacteriocin gene clusters using BAGEL4 software, with 339 gene clusters of interest being identified. Class I was the most abundant bacteriocin class predicted, accounting for 91.3% of predictions, Class III had a predicted abundance of 7.5%, and Class II was represented by just 1% of all predictions.

## 1. Introduction

Physical fitness has been associated with a better quality of life and, in general, fewer reported days of illness [1]. Exercise has also been shown to have beneficial effects concerning risk reduction of cardiovascular disease [2], anti-inflammatory potential [3], mental health including depression [4], and microbiome modulation [5,6,7,8,9].

The intestinal microbiome is a rich and diverse ecosystem collectively composed of 100 trillion cells, including bacterial, fungal, viral, and archaeal cells [10], which can cooperate/compete with each other and the host [11]. In the last decade, it has been well-documented that athletes have a more diverse microbiome when compared to non-athletes, often associated with differences in the relative abundance of certain bacterial taxa, including but not restricted to *Akkermansia, Faecalibacterium, Prevotella,* and *Veillonella* [5,8,9,12]. There is some evidence that these changes in the athlete microbiome arise as a result of a long-term adaptation, as opposed to a short-term exercise intervention. Indeed, Cronin and colleagues investigated the impact of an 8-week exercise regime and found that changes in the microbiome were subtle [6]. These recent findings have led us to hypothesise that the gut microbiome of elite athletes could be a possible source of antimicrobial peptide (AMP) producing bacteria and could potentially be exploited to harness bacteria with potential novel functions and probiotic traits (i.e., bacteriocin production).

Bacteriocins are small, heat-stable, ribosomally-synthesised antimicrobial peptides produced by one bacteria that are active against other bacteria, to which the producer strain is immune [12]. Antimicrobial peptides and especially bacteriocins have received increasing interest due to their potential applications in the treatment of bacterial infections owing to the growing threat of antimicrobial resistance (AMR). Bacteriocins and bacteriocin-producing bacteria are promising tools regarding preventing or treating target bacterial infections as many bacteriocins have a narrow spectrum of activity, ergo causing minimal disruption to the microbiome as a whole [13,14]. Indeed, bacteriocin production can be regarded as a desirable probiotic trait as it can aid in (a) inhibiting the growth of various pathogens in the gut [15], (b) support the colonization of desirable species in the gut [16], and (c) act as signaling peptides through quorum sensing systems [17]. Quorum sensing systems play a vital role in biofilm formation, which could prolong the resident time of probiotic bacteria in the gut [18] and therefore influence the host’s health. Past studies have shown that bacteriocins are attractive alternatives to antibiotic treatment [19,20,21], and such therapies should be further studied. Bacteriocin functionality is reliant on several genes working in tandem. At a minimum, a functional bacteriocin gene cluster needs a structural gene and an immunity gene (to protect the bacteriocin producer strain) [22,23].

The intestinal microbiota is one of the richest storehouses for bacteriocin-producing bacteria. Two previous data mining projects have investigated the prevalence of bacteriocins in the human gut. Both studies have identified bacteriocin gene clusters within the Firmicutes, Proteobacteria, Bacteroides, and Actinobacteria phyla, highlighting the abundance and diversity of bacteriocins in the gut [24,25]. Another study investigated bacteriocin diversity on several body sites, including the gut microbiome. They found that the gut had a high abundance of bacteriocin producers from the following species; *Bacteroides fragilis*, *Bacteroides dorei*, *Eubacterium rectale*, *Escherichia coli,* and *Blautia hansenii* [26]. Previous work in the literature has shown the applications of bacteriocins in controlling important pathogens. A murine study by Corr et al. found that a *Lactobacillus salivarius* UCC118 gut isolate produced an Abp118 bacteriocin, which protected the mice against a *Listeria monocytogenes* infection through direct antagonism [16]. Similarly, Bacteriocin 21, produced by a gut commensal *Enterococcus faecalis*, has been shown to inhibit the growth of vancomycin-resistant enterococci in a mouse model [27]. Other in vitro studies have also demonstrated the impact of bacteriocin-producing strains on known human pathogens, namely *Clostridioides difficile* [28], *Salmonella* spp. [16,29], *Enterococcus faecium* [30], *Helicobacter pylori* [31], *Bacillus cereus* [32] and *Campylobacter* spp. [33].

**Figure 1:**
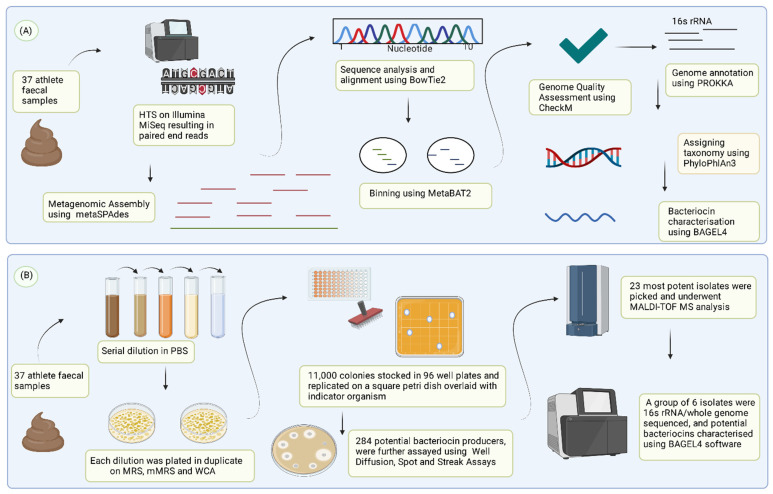
In silico and in vitro based approaches used in this study to identify potential novel bacteriocins from the athlete’s gut. (**A**) Metagenomic data from 37 faecal samples in the form of paired-end reads were assembled, annotated, quality-checked, and binned to recover Metagenome-Assembled Genomes (MAGS) analysed using BAGEL4 for the presence of potential bacteriocin genes. (**B**) 37 faecal samples from elite Irish athletes were screened for novel bacteriocin-producing gut isolates. Potential bacteriocin producers were assayed further, and the spectrum of inhibition was assessed. Isolates exhibiting potential antimicrobial activity were brought forward for MALDI-TOF mass spectrophotometry, whole-genome sequencing (WGS), and bacteriocin biosynthetic gene clusters were predicted using BAGEL4 software. (Figure created with https://BioRender.com, accessed on 15 February 2022).

For the purpose of this study, we selected two important gut pathogens, *Fusobacterium nucleatum* and *C. difficle,* as our indicator strains to mine the athlete gut microbiome for producers of antimicrobials. *C. difficle* is a Gram-positive [34] enteric pathogen causing *C. difficile*-associated diarrhoea (CDAD) [35]. It has also been shown to have the ability to disrupt the gut microbiota of colonised persons [36]. It has become clear that novel treatment options should be sought, with one study suggesting the mortality rate of CDAD can reach as high as 25% in the elderly populations [37]. It has been shown to affect both the elderly and younger immunocompromised populations [38]. *F. nucleatum* is a Gram-negative bacterium [39] associated with several intestinal pathologies, including colorectal cancer (CRC) development and progression [40]. There is considerable merit in identifying antimicrobials that could contribute to novel treatment options to control these targets. This study aimed to screen the microbiome of an Irish athlete cohort for potential novel bacteriocins, using both in vitro and in silico based approaches. Our in silico approach looked at the reconstruction of Metagenome Assembled Genomes (MAGs) from the sequenced faecal samples, followed by predicting bacteriocin gene clusters present within the assemblies using the BAGEL4 software. We have then used athlete faecal samples associated with the recovered MAGs to screen the gut microbiome of an athlete cohort for novel bacteriocin producers in vitro (Figure 1).

## 2. Materials and Methods

### 2.1. Subject Recruitment and Sample Collection

An existing bank of elite athlete faecal samples was used in this study (O’Donovan et al. [7]). The recruitment criteria were as follows: Irish athletes/athletes representing Ireland, preparing for and/or participating in international competitions (including the Olympics). The ethical approval for the study was granted by the clinical research ethics committee (Project code: APC073). All subjects gave written informed consent before the study. Stool samples were collected from male (*n* = 23) and female (*n* = 14) athletes and stored anaerobically at −80 °C prior to culture-based analysis.

### 2.2. Recovery of Metagenome Assembled Genomes (MAGs) and Antimicrobial Peptide Production Analysis

Metagenomic data from 37 faecal samples in the form of paired-end reads were obtained from a previous study [7]. The raw data are available in the European Nucleotide Archive (ENA) under the accession number PRJEB32794. Human reads were removed with BMTagger [41], the resulting shotgun fastq files were converted to BAM files using Picard Tools (http://broadinstitute.github.io/picard/). The BAM files were then quality trimmed and duplicates removed using SAMTools v1.9 [42]. Metagenome assembly was performed de novo using MetaSPAdes assembler 3.13 [43], followed by sequence analysis and alignment using BowTie2 v.2.3.4 [44]. Genome binning was performed using MetaBAT2 [45]. The quality (completeness and contamination) of constructed MAGs was determined using CheckM [46]. MAGs with <90% Completeness and >5% Contamination were deemed Low-Quality and were dismissed from further analysis, while those with >90% Completeness and <5% Contamination were deemed High-Quality and were brought forward for further analysis [47]. High-Quality MAGs were annotated using PROKKA v.1.13 [48] and assigned taxonomy with PhyloPhlan3 v.3.0, SGB.Dec19 database, using the default 5% as the assignment threshold [49]. The presence of antimicrobial peptide gene clusters was assessed using BAGEL4 [50]. A gene set was considered a putative bacteriocin gene cluster if it contained a minimum of: transport/immunity gene, modification gene (for post-translationally modified peptides), leader-cleavage peptide, and a structural peptide [23].

### 2.3. Isolation of Bacterial Isolates Producing Antimicrobial Peptides

For this study, culturing conditions were used to target the isolation of bifidobacteria, lactobacilli, and culturable gut anaerobic species, whereby one gram of frozen faecal sample was suspended in Phosphate Buffered Saline (PBS) and serially diluted ten-fold. Each dilution was spread-plated onto various selective agars with a final agar concentration of 1.5% (*w*/*v*). Bifidobacteria were isolated anaerobically on De Man, Rogosa, and Sharpe agar (MRS; BD^TM^ Difco^TM^ Trafalgar Scientific Ltd., Leicester, UK) supplemented with 0.05% (*w*/*v*) L-cysteine hydrochloride (Sigma, London, UK) (noted as mMRS agar). mMRS agar was further supplemented with Mupirocin (Sigma, London, UK) at 200 µg/mL of medium. *Lactobacillus* species were isolated on LBS agar (BD^TM^ Difco^TM^ Trafalgar Scientific Ltd.,Leicester, UK) aerobically and anaerobically. Obligate anaerobic species were isolated on Wilkins-Chalgren Media (Sigma, London, UK) in an anaerobic chamber. Plates were incubated at 37 °C for 24–48 h. The isolates were sub-cultured in their respective liquid growth medium with 10% (*v*/*v*) glycerol and stored in 96 well plates at −80 °C.

### 2.4. Antimicrobial Activity Assays

Frozen bacterial stocks were replicated into 96 well plates containing the relevant liquid growth medium using a multi-pin stamper (Boekel Scientific, Feasterville-Trevose, PA, USA) and incubated at 37 °C for 24–48 h anaerobically. Liquid cultures were then sub-cultured by replication onto large petri dishes containing the corresponding growth medium solidified with 1.5% (*w*/*v*) agar and incubated for 24–48 h until isolated colonies were visible. Petri-dishes were overlaid with growth medium solidified with agar (0.8% *w*/*v*) seeded with 1% overnight inoculum of different indicator strains. The indicator strains and their respective growth conditions are summarised in Table 1. Colonies showing possible bacteriocin activity were selected for further characterisation.

### 2.5. Characterisation of Antimicrobial Activity of Putative Bacteriocin-Producers

#### 2.5.1. Well Diffusion Assay (WDA)

Pure cultures of potential bacteriocin-producers were obtained by inoculating 10 mL of a sterile liquid medium with frozen stock cultures and incubating for 24 h. Cell-free supernatant (CFS) was prepared from 2 mL of overnight culture by centrifugation for 3 min at 14,000 rpm. Wells were made in agar plates containing appropriate growth medium solidified with agar (0.8% *w*/*v*) and seeded with overnight cultures of indicator strains (200 µL inoculum/20 mL soft media). 50 µL of CFS of the putative bacteriocin-producing strain was pipetted into the well. Plates were left to dry and incubated overnight [51]. Zones of inhibition around the wells were assessed. Strains exhibiting antimicrobial activity were kept for further investigation and were genetically characterised using 16S rRNA sequencing and/or molecular masses of the active peptides were confirmed using MALDI-TOF MS. Peptide masses were compared with the Bactibase online database (http://bactibase.hammamilab.org/main.php, accessed on 2 June 2021) [52].

#### 2.5.2. Identification of Putative Bacteriocin-Producing Strains Using 16S rDNA Analysis

Genomic DNA was extracted from 10 mL of liquid culture using a Qiagen PowerFaecal Pro DNA extraction kit (Qiagen, United Kingdom). For PCR reactions, Platinum Master Mix (Fisher Scientific, Ireland) was used with universal bacterial primers CO1 5′-AGTTTGATCCTGGCTCAG-3′ and CO2 5′-TACCTTGTTACGACT-3′ (PCR run conditions: 94 °C × 2 min (×1 cycle), 94 °C × 30 s, 52 °C × 30 s, 72 °C × 1 min (×30 cycles), 72 °C × 5 min (×1 cycle)). PCR reactions were purified using the Qiagen PCR Cleanup Kit (Qiagen, Manchester, UK). Sequencing of the amplicons was performed by GATC Biotech (Koln, Germany). Species designation was carried out using the 16S ribosomal RNA sequences database on the Basic Local Alignment Search Tool (BLAST), using >97% sequence identity.

#### 2.5.3. Shotgun Whole-Genome Sequencing (WGS) and Analysis

Genomic DNA was extracted from 10 mL of overnight liquid cultures using GeneElute™ Bacterial Genome DNA Kit (Sigma-Aldrich, Arklow, Ireland). The concentration of extracted DNA was confirmed using a Qubit^®^ 2.0 Fluorometer (ThermoFisher Scientific, Dublin, Ireland) according to standard protocols, and samples were then standardised to 0.2 ng/µL of DNA. Standardised DNA was then prepared for whole-genome sequencing using the Nextera XT DNA protocol (Illumina, San Diego, CA, USA), using their standard protocol guide and sequenced on Illumina NextSeq platform following standard Teagasc protocols. The paired-end reads underwent quality control using trim_galore (https://www.bioinformatics.babraham.ac.uk/projects/trim_galore/) and assembly into contigs using the SPAdes v.3.13 [53] software, using default settings, genes were predicted and annotated using PROKKA v.1.13 [48]. The assembled contigs were analysed using BAGEL4 [50] to assess antimicrobial activity, taxonomy assignment was performed using the *atpA* gene and confirmed using the GTDB-Tk software v.1.3 [54]. Raw sequence reads are available under the accession number PRJEB48530. Antimicrobial genes present were identified using the CARD Resistance Gene Identifier (RGI) database [55].

#### 2.5.4. MALDI-TOF Mass Spectrometry

Single colonies of each strain were inoculated into 5 mL volumes of MRS broth and incubated at 37 °C overnight. 250 µL of each inoculum was used to inoculate 25 mL volumes of MRS, which were in turn incubated at 37 °C overnight. 50 µL aliquots of each cell-free culture supernatant (CFS) were plated on *Lactobacillus bulgaricus* and *Listeria innocua* indicator plates. The 25 mL inocula were used to inoculate 600 mL volumes of XAD MRS (MRS passed through a column containing XAD) and MRS and incubated as described. Individual cultures were centrifuged at 11,000× *g* for 20 min, and cells were separated from supernatant. Cells were mixed with 150 mL 70% propan-2-ol and stirred at room temperature for 3–4 h. The cell extract was centrifuged again, and the supernatant was retained for purification attempts. MALDI-TOF analysis was undertaken on strains of interest.

### 2.6. Targeted Assembly of Metagenome Assembled Genomes

Five *Enterococcus* species (*E. faecalis, E. faecium, Enterococcus durans, E. mundtii,* and *Enterococcus hirae*) commonly found in the human gut [56] were selected, and representative genomes were downloaded from RefSeq. We downloaded 1866 *E. faecalis*, 2374 *E. faecium,* 124 *E. durans,* 48 *E. mundtii,* and 386 *E. hirae* genomes.

Five separate reference genome databases were created for each bacterial species mentioned above. Assembled contigs were blasted against each reference database, and output was filtered to ascertain the contigs that aligned with the database, with >95% identity and >50% coverage. The faSomeRecords.py script (downloaded from: https://github.com/santiagosnchez/faSomeRecords, accessed on 9 November 2021) was used to extract the aligned contigs and subsequently convert them into a single multifasta file to represent a single MAG. The MAGs were then genome quality assessed using the CheckM software [46], where a threshold of Completeness and Contamination was set to >90% and <5%, respectively.

### 2.7. Statistical Analysis

Multidimensional Scaling analysis of Bray-Curtis distance was performed on metagenomic data (paired-end reads) from 37 Irish athletes [7] and 21 Low BMI controls previously used in a study by Barton et al. [57]. Raw Reads from Barton et al. are available under the accession number PRJEB15388. Statistical analysis and figure visualization were performed in RStudio 3.0.1, using the following packages “ggplot2”, “vegan”, “reshape”, “harry potter”, and “dplyr”.

## 3. Results

### 3.1. Assessment and Recovery of Metagenome Assembled Genomes (MAGs) for Bacteriocinogenic Potential

**Figure 2:**
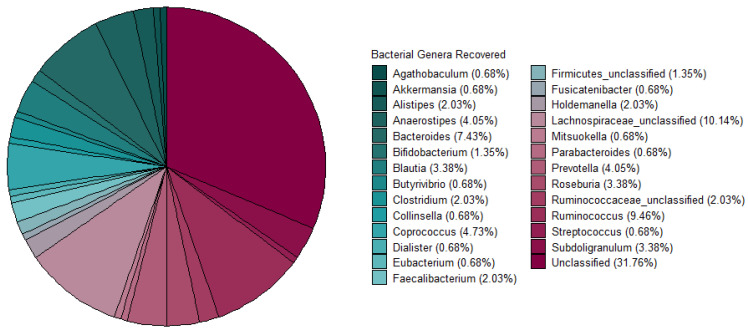
Bacterial genera recovered from Metagenome Assembled Genomes. The majority of MAGs were unclassified, followed by a high abundance of; *Lachnospiraceae_unclassified*, *Bacteroides*, *Ruminococcus,* and *Coprococcus,* using PhyloPHhlAn3 to assign taxonomy.

**Figure 3:**
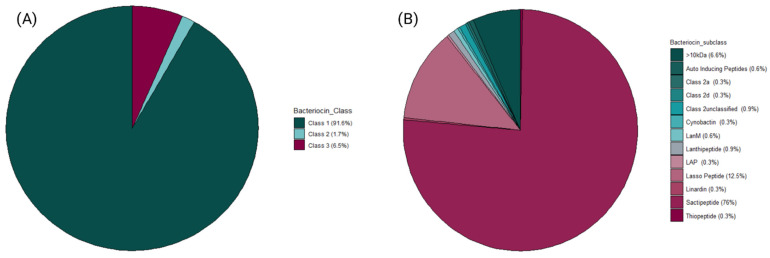
Frequency of bacteriocin classes and their subsequent t subclasses predicted by BAGEL4 from the recovered MAGs. Class I, II, and III bacteriocins were predicted, with Class_1 (including Sactipeptide sub-group) found to be the most abundant predicted bacteriocin class within the athlete gut sampled in this study. Sactipeptides and Lasso peptides were amongst the most predicted bacteriocin sub-classes, followed by >10 kDa. (**A**) Contains all bacteriocin classes predicted by BAGEL4 software (**B**) Contains all bacteriocin sub-classes predicted by BAGEL4 software.

Metagenomic sequencing data from 37 faecal samples (obtained from [7]) yielded 770 MAGs in total. For this study, 148 High-Quality MAGs (>90% Completeness and <5% Contamination) were used and assigned taxonomy using PhyloPhlAn3. A majority of the MAGs were unclassified at the genus level after taxonomic assignment. The most abundant genera recovered were *Lachnospiraceae_unclassified*, *Bacteroides*, *Ruminococcus,* and *Coprococcus* (Figure 2). The bacteriocinogenic potential of the MAGs recovered from the athlete faecal metagenomic data was then assessed using BAGEL4. The data shows that 91.3% of predicted bacteriocins corresponded to Class I bacteriocins, followed by representatives of Class III (antimicrobial proteins >10 kDa in mass), and 7.5% and 1% to Class II (Figure 3A). The sactipeptide sub-class was particularly abundant, representing 76% of the predicted Class I bacteriocins; Lasso peptides were the second most abundant sub-class (12%) with the remaining sub-classes predicted at >1% abundance (Auto-Inducing Peptides, LanM, Thiopeptide, Lanthipeptides, Linardin, Cyanobactin, and LAP bacteriocins). Class III was represented by Zoocin A-like clusters (90% of predicted Class III bacteriocins) and Closticin_574. Lastly, the Class II group of predicted bacteriocins consisted of Class IIa, Class IId and Class II unclassified (Figure 3B).

### 3.2. Detection of Bacteriocin-Producing Bacterial Isolates from Athlete Faecal Samples

Due to the high abundance of anaerobic species recovered in our in silico analysis, we decided to culture these using WCA agar (Figure 2). We also observed bifidobacteria genus MAGs in our samples and therefore decided to use mMRS agar for isolation. Under this approach, approximately 11,000 colonies of different morphologies were isolated from 37 athlete faecal samples. The samples used in this study were from elite athletes who were preparing to partake in international competitions (including the Rio Olympics [7]). mMRS agar with added mupirocin was used as it was previously shown to successfully isolate *Bifidobacterium* spp. from faecal samples [58,59], and approximately 3500 presumptive bifidobacteria were isolated. BD LBS Agar was employed to isolate 1500 presumptive *Lactobacillus* isolates, and finally, obligate anaerobic species (6000 isolates) were recovered on WCA, which is commonly used for the isolation and enumeration of anaerobic species [60,61]. Gut isolates stocked in 96-well plates were replicated onto agar plates, and the resulting colonies were screened for antimicrobial activity using a soft agar overlay seeded with indicator strains. In several instances, putative antimicrobial activity was displayed by a majority of isolates, thus precluding identification of individual producer strains. To circumvent this, isolates that showed the most potent antimicrobial activity were selected, and well assays were performed. This resulted in a total of 284 potential bacteriocin-producing gut isolates being identified that were active against at least one of the four indicator strains used. Initially, isolates recovered on MRS and mMRS were screened for activity against *L. innocua* and *L. bulgaricus*. However, the screening detected just one colony with antagonistic activity against *L. bulgaricus*, representing a very low isolation frequency of 0.02%. Similarly, a very low isolation frequency (0.04%) was noted for isolates with antimicrobial activity against *L. innocua*. Subsequent use of WCA medium to recover gut isolates exhibiting antimicrobial activity against *C. difficile* and *F. nucleatum* yielded higher isolation frequencies of 2.4% (*C. difficile*, 145 isolates) and 2.26% (*F. nucleatum*, 136 isolates) (summarised in Table 2).

### 3.3. Identification of Putative Bacteriocin-Producing Strains Isolated from Faecal Samples

Gut isolates exhibiting putative bacteriocin activity were narrowed down further based on the size of the zone produced, and the six most promising gut isolates, recovered from either mMRS or WCA, were brought forward for further characterisation (summarized in Table 3). We recovered three isolates from mMRS showing putative antimicrobial activity against *L. innocua* and/or *L. bulgaricus*. We also brought forward the three most potent isolates recovered from WCA with antagonistic activity against *F. nucleatum* and/or *C. difficile* (see Table 3).

The three mMRS-recovered gut isolates were identified using shown to be *Enterococcus* species based on 16S rRNA Sanger sequencing (see Table 3). We also endeavoured to determine the mass of the putative bacteriocins produced by each isolate using MALDI-TOF MS, compared them with the Bactibase online database, and all three masses aligned with well-characterised bacteriocins (see Supplementary Figure S1). The bacteriocin produced by isolate LW003 showed antagonistic activity against *L. bulgaricus* and had a molecular mass of 5207 Da and 5218 Da, which corresponds to the individual components of the two-peptide bacteriocin, enterocin 62-6 of the class IIc bacteriocin classification group [49]. The other two enterococci isolates, LW001 and LW002, exhibited activity against *L. innocua* and were found to produce molecules with masses of 3979 Da and 3977 Da, respectively, which correlate to the previously characterised enterocin Q, a leaderless class IIc bacteriocin [50]. The three aforementioned gut isolates were not brought forward for whole-genome sequencing due to the high occurrence of antagonistic activity against *C. difficile* and/or *F. nucleatum*, which held a greater interest for the context of the project.

**Figure 4:**
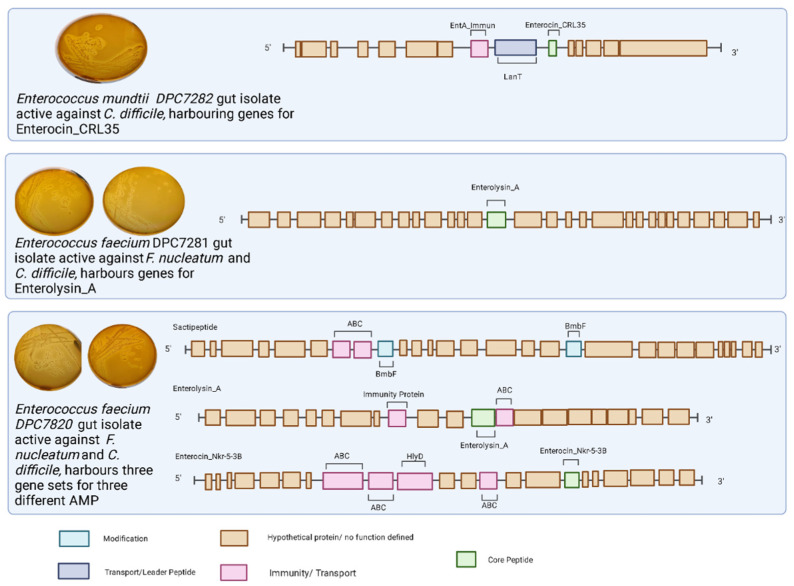
BAGEL4 outputs for gut isolates active against *F. nucleatum* and/or *C. difficile***.** Three bacterial isolates recovered from WCA underwent whole-genome sequencing and taxonomy assignment using GTDB-Tk software. Putative bacteriocin gene clusters were annotated and predicted using the BAGEL4 software. Figure created with (https://BioRender.com, accessed on 15 February 2022).

As noted above, we selected the three most potent WCA isolates for further analysis and subjected these were subjected to whole-genome sequencing. The two anti-*Fusobacterium nucleatum* gut isolates (DPC7280 and DPC7281) were identified as *E. faecalis,* and the anti-*Clostridioides difficile* isolate (DPC7282) was assigned as an *E. mundtii* (see Table 3). The two *E. faecalis* gut isolates active against *F. nucleatum* possessed a putative gene cluster corresponding to the class III bacteriocin enterolysin A [62]. In addition to enterolysin A, strain DPC7280 harbours bacteriocin gene clusters predicted to encode enterocin Nkr-5-3b and a potentially novel functional sactipeptide operon (see Figure 4). The predicted sactipeptide operon carries an ABC transporter permease, a protein often associated with bacteriocin transportation across the membrane [52], ABC transporter binding protein possibly associated with self-immunity [53], and a SPASM domain-containing protein, which could be involved in peptide modification [54], however, a structural gene was not be identified (see Figure 4). Finally, the *E. mundtii* isolate (DPC7282) harbours a gene cluster corresponding to that which encodes enterocin CRL35 bacteriocin, belonging to a class IIa bacteriocin with demonstrated activity against *Listeria* species [55]. The molecular masses corresponding to bacteriocins encoded by these clusters were not detected through colony mass spectrometry analysis (see Supplementary Figure S1).

### 3.4. Assessment of Potential Bacteriocin-Producing Strains for Antimicrobial Resistance Genes (ARGs)

The three isolates active against *C. difficile* and/or *F. nucleatum* were assessed for the presence of ARGs using the CARD RGI database (Table 3). Both *E. faecalis* isolates DPC7280and DPC7281 were found to harbour genes for *dfrE* and *efrA*. Additionally, isolate DPC7281 was found to carry six additional ARG genes; tetM, ErmB, *E. faecalis* chloramphenicol acetyltransferase, *aad*(6), SAT-4, and APH(3′)-IIIa. Isolate *E. mundtii* (DPC7282) did not contain any ARGs.

### 3.5. Targeted Assembly of Metagenome Assembled Genomes (MAGs)

Due to the overwhelming recovery of *Enterococcus* spp. isolates in the in vitro screen, we subsequently specified our MAG assemblies to target the *Enterococcus* genus. In this targeted bioinformatics approach, contigs obtained from our metagenomic assembly were blasted against our five different reference databases, which represent the five species of interest (*E. faecalis, E. faecium, E. durans, E*. *mundtii,* and *E. hirae*). We have chosen these *Enterococcus* species of interest in consideration of the most frequent and abundant *Enterococcus* spp. associated with the human gut microbiome [43]. Results are presented in the form of BLAST hits (see Table 4/see Supplementary Figure S2). Metagenomic contigs aligning to the *E. faecium* reference database numbered 670,970,682 BLAST hits, followed by *E. faecalis* with 73,372,776 hits recovered, *E. mundtii* with 2,732,290 hits recovered, *E. hirae* with 13,166,269, and finally, *E. durans* with 9,623,616 hits. We recovered 40 bins/MAGs for each species of interest, resulting in 200 bins/MAGs recovered. A set of two *E. faecalis* MAGs were recovered from the low-quality MAG category (<30% Completeness, <10% Contamination); the remaining 198 MAG bins possessed <13% Completeness and therefore could not be assigned to any quality group (see Supplementary Excel S1).

## 4. Discussion

This study is one of the first to target the athlete gut microbiome, a high diversity niche for a potential source of novel antimicrobial agents. In this study, we aimed to identify AMP-producing strains in the athlete’s gut. Elite athletes and their associated microbiomes could be viewed as potentially different and more diverse than the general population [5,8,12]. We have also verified this using healthy controls and beta diversity measures. (see Supplementary Figure S3).

The combined use of in vitro and in silico approaches allowed for a broader investigation of the bacteriocinogenic potential of the athlete gut. Our in silico analysis recovered a large abundance of anaerobic gut species, often associated with the athlete gut microbiome (i.e., *Bacteroides* spp. [63], *Collinsella* spp. [64,65], *Coprococcus* spp. [66,67], *Eubacterium* spp. [8], *Prevotella* spp. [8] and *Ruminococcus* spp [8,62,65,66]. (Figure 2)). For the purpose of re-isolation of the aforementioned species, we have decided to use WCA agar, widely used in isolation of strict anaerobic gut species.

We have also predicted the presence of a myriad of bacteriocin classes and sub-classes embedded in the metagenome-assembled genomes; Class I bacteriocins were particularly abundant at 91%, out of which 76% of all predictions belonged to sactipeptides. This agrees with a previous study by Walsh et al. [25], where they found a high abundance of sactipeptides within the human gut microbiome. Class III bacteriocins were abundant at 7.5% of all bacteriocins, and Class II bacteriocins comprised just 1% of all predictions.

It is interesting to note that all the bacteriocin-producing gut isolates brought forward for further analysis in this study were found to be *Enterococcus* spp. *Enterococcus*-selective media was not used in the present study; there is no evidence in the literature to support the increased abundance of *Enterococcus* spp. in athlete cohorts [5,8,9,12], quite on the contrary—one study found *Enterococcus* spp. to be decreased within the exercise group of a murine model [68]. It is possible that the rich composition of Wilkins-Chalgren medium (WCA) combined with the generally non-fastidious requirements of members of the *Enterococcus* genus allowed for their overgrowth and subsequent overrepresentation of the *Enterococcus* spp. in the library of culturable isolates.

The frequency of isolation of strains with activity against the indicators *L. innocua* and *L. bulgaricus* is comparable to that for previous studies [69,70], however, the frequency of isolation observed for *C. difficile* appears to be higher than current observations in the literature [71]. Screening of gut isolates against *F. nucleatum* has not yet been addressed, and therefore no data exists for direct comparison.

As noted, it is possible that *Enterococcus* spp. overgrew their commensal counterparts during culturing and are subsequently overrepresented within the biobank community of isolates. This could be attributed to the fact that obligate anaerobic species were isolated on WCA, an agar used to isolate a wide variety of anaerobic species. This combined with the fact that *Enterococcus* spp. are generally less fastidious than other anaerobic microbiome commensals, and could potentially attribute to the subsequently higher isolation of *Enterococcus* spp. isolation. We also suspect a high incidence of repeated isolation of the *Enterococcus* species with activity against *C. difficile* and/or *F. nucleatum*, which could explain the higher isolation frequency observed with the aforementioned enteric pathogen indicators.

In contrast, *Bifidobacterium* spp. and *Lactobacillus* spp. were isolated on genus-specific culture media, leaving little room for *Enterococcus* spp. to dominate the culturing environment. Nevertheless, our observations support the possibility that the *Enterococcus* spp. isolated in this study tended to exhibit strong antagonistic activity against *C. difficile* and/or *F. nucleatum*.

The *Enterococcus* spp. isolates with activity against *L. innocua* (LW001 and LW002) and/or *L. bulgarcius* (LW003) had molecular masses that corresponded to those of enterocin Q and enterocin 62-6, respectively, which is not surprising as enterocin Q is known to inhibit species of the *Listeria* genus [72]. Similarly, enterocin 62-6 has been demonstrated to inhibit Gram-positive bacterial species [73].

This is, to our knowledge, the first report of an *E. mundtii* gut isolate harbouring the gene cluster for enterocin CRL35 showing activity against *C. difficile.* Enterocin CRL35 displayed strong antagonism against *C. difficile* and little activity against *F. nucleatum*, implying it has a narrow spectrum. This aligns with previous studies that illustrated the narrow spectrum usually observed in Class IIa bacteriocins (reviewed by [74]). A literature search presented limited information regarding the functionality of the bacteriocin; however, its ability to inhibit the gut pathogen *Listeria monocytogenes* has been well documented [75]. However, the ability of *Enterococcus* spp. to inhibit the growth of *C. difficile* has been well-documented. Bacteriocin biosynthetic gene clusters corresponding to those for duracin 61A, enterocin AS-48, enterocin A/B/P, and Q amongst others (as reviewed by [76] have all been noted to possess antimicrobial activity against *C. difficile.*

Additionally, we also show for the first time that an *E. faecium* gut isolate harbouring genes encoding enterolysin A inhibits *F. nucleatum*, a gut pathogen associated with colorectal cancer [77]. This would appear to support the findings of a recent human trial investigating the administration of a multi-strain probiotic cocktail including *E. faecalis* in colorectal cancer patients, where a 5-fold decrease in *F. nucleatum* was observed in the probiotic-supplementation group of the study [78]. Enterolysin A is known to inhibit the growth of several enterococci, pedicocci, lactococci, and lactobacilli [62,79], as well as *Listeria, Bacillus,* and *Staphylococcus* species [62]. Similarly, a second *E. faecium* isolate displayed antagonistic activity against both *F. nucleatum* and *C. difficile*. BAGEL4 predicted the presence of three biosynthetic gene clusters, a putative sactipeptide, enterolysin A, and enterocin NKR-5-3B. Sactipeptides have previously been shown to inhibit the growth of *C. difficile* and may contribute to this isolates activity [80]. Enterocin NKR-5-3B is a circular bacteriocin that displays a broad spectrum of activity, inhibiting a wide range of Gram-positive species [81]. The presence of the enterocin NKR-5-3B gene cluster in the genome of the enterococcal gut isolate DPC7280 may account for its inhibitory activity against *C. difficile*. *Enterococci* spp. bacteriocins are well known for their inhibitory activity against *Listeria* spp. Previous studies demonstrated the ability of helveticin [82], hiraecin S [83], enterocin 1146 [84], and bacteriocins RZS C5 and RZS C13 produced by *E. faecium* [85] to all have antimicrobial activity against *Listeria* spp. strains.

We have also assessed the AMR profile of the sequenced genomes active against *F. nucleatum* and/or *C. difficile*. In recent years enterococci have become resistant to many commonly used antibiotics, i.e., erythromycin and tetracycline [86], and therefore, interest in its antimicrobial resistance profile is of growing importance. Interestingly, *E. mundtii* DPC7282 strain did not contain any ARGs. *E. mundtii* usually carries a less significant ARG profile when compared to other members of the enterococci genus [87], with some studies reporting no ARGs present within the *E. mundtii* genomes [88,89]. Isolates DPC7281 and DPC7280 were resistant to diaminopyrimidine (*dfrE*) and possessed a multidrug efflux pump (*efrA*). Additionally, isolate DPC7281 was found to carry resistance genes to six additional antibiotics; tetracycline (*tetM*), macrolides (*ErmB*), chloramphenicol (*E. faecalis* chloramphenicol acetyltransferase), aminoglycosides (*aad(6)* and *APH(3′)-IIIa*), and streptothricin (*SAT-4*). The presence of the aforementioned genes has been previously noted in the literature as common resistance mechanisms in enterococci genomes [90,91,92,93,94,95,96,97,98].

Initial MAG recovery did not yield any genomes assigned to the *Enterococcus* genus. We then specified our genome assembly using a targeted binning approach. Even though we have not recovered any bins/MAGs that matched our threshold (Completeness > 90%, Contamination < 5%), we have recovered millions of alignments/hits corresponding to the targeted *Enterococcus* spp. of interest, which are commonly found in the human gut. These results confirm that reads corresponding to *Enterococcus* spp. are present in the microbiome of elite athletes. However, due to a suspected low abundance of enterococci in the athlete gut, we did not recover any high-quality MAGs corresponding to the genus [68]. Our findings are substantiated by recent studies concluding that *Enterococcus* spp. genomes are difficult to assemble and recover from metagenomic samples due to high gene divergence and high genome plasticity [99]. *E. faecalis* in particular has been shown to have high levels of genome plasticity, insertions/deletions and repetitive regions, which can hinder successful assembly [99,100].

This study highlights the merits and disadvantages of both in silico and in vitro based approaches. In silico screening allowed for a broader representation of the taxonomical and functional composition of the niche at hand, without the inherent bias introduced by culturing microorganisms present in the samples. Conversely, in vitro approaches allowed for the isolation of bacterial species and relatively rapid assessment of their clinical relevance using established assays for antagonistic activity against a selection of important enteric pathogens. Ultimately, both approaches contain distinct inherent biases. In silico analysis, in this instance the BAGEL4 tool, is reliant on homology and can predict spurious matches on that basis. Another important drawback is that in silico evaluation of metagenomic datasets, particularly the presence of potential antimicrobial gene clusters as in the present study, is dependent on the degree of success of assembly of the metagenome, which can vary according to the genomic composition of constituent species. Similarly, the isolation and in vitro screening process is influenced by the choice of indicator species for antagonistic assays, antimicrobial expression conditions of certain microbial taxa, including environmental and other microbial and host factors, and the degree to which a species exhibits obligate or facultative growth in vitro. These dynamic factors are not apparent in in silico analysis yet may contribute to false negatives in the isolation pipeline as well as the overrepresentation of certain species in the pool of isolates. It is also vital to recognize that identified isolates with potential antimicrobial activity require verification of the mode of action, for instance, through cloning and functional expression of putative bacteriocin genes or purification/direct chemical synthesis.

## 5. Conclusions

In conclusion, the gut microbiome of the elite athletes in this study appeared to be a rich source of AMPs with potential applications in human health.

*In silico* approaches can be used to provide a broad overview of the bacterial taxa present and their potential metabolites, which can inform the design of the in vitro screen. Similarly, in vitro results can also validate in silico results, as shown in the present study through an *Enterococcus*-specific approach.

Our in silico analysis identified a broad range of potential bacteriocin classes present in the athlete gut, suggesting the athlete gut could be used to harness novel natural bacteriocin-producers for potential development as alternatives to existing antibiotics.

Putative bacteriocin-producing gut isolates identified in this study through in vitro analysis could be harnessed as an alternative treatment against relevant gut/enteric pathogens (*F. nucleatum* and *C. difficile),* especially *E. mundtii* isolate, which was shown to harbour no ARGs.

Therefore, we suggest a tandem deployment of in silico and in vitro approaches to broadly interrogate the niche at hand.

## Figures and Tables

**Table 1:** Bacterial indicators used in this study and their respective growth conditions.

Bacterial Strain	Media for Cultivation	Growth Atmosphere	Temperature (°C)
*Lactobacillus bulgaricus* LMG6901 *	MRS	Anaerobic ******	37
*Listeria innocua* DPC3572 **	BHI ***	Aerobic	37
*Fusobacterium nucleatum* DPC6999	FAA/WCA ****	Anaerobic	37
*Clostridioides difficile* DPC6509	RCA/BHI *****	Anaerobic	37

* LMG = Belgian Co-ordinated Collections of Microorganisms. ** DPC = Teagasc Culture Collection *** BHI = Brain Heart Infusion (Sigma, London UK) **** FAA/WCA = Fastidious Anaerobic Agar/Wilkins Charlgreen Agar (LabM, Bury UK/ Sigma, London UK) ***** RCA = Reinforced Clostridial Agar (Sigma, London UK) ****** anaerobic conditions were achieved using an anaerobic chamber, except for *L. bulgaricus,* for which anaerobic jars and Anaerocult gas packs (Merck, Darmstadt, Germany) were used.

**Table 2:** Isolation frequency of intestinal gut isolates using different indicator organisms.

Indicator Organism	Number of Isolates Screened	Isolates with Antagonistic Activity against Indicator	Frequency of Isolation %
*Listeria innocua* DPC3572	5000	2	0.04%
*Lactobacillus bulgaricus* LMG6901	5000	1	0.02%
*Fusobacterium nucleatum* DPC6999	6000	136	2.26%
*Clostridioides difficile* DPC6509	6000	145	2.42%

**Table 3:** Most promising gut isolates showing antagonistic activity against indicators of choice.

Strain Designation	Media	Indicator	Taxonomy Method	Genus/Species	Method of Bacteriocin Prediction	Bacteriocin Predicted	AMR Genes Identified
LW001	mMRS × 2 [Mupirocin]	*Listeria innocua*	16s rRNA	*Enterococcus*	MALDI-TOF	Enterocin Q	n/a
LW002	mMRS × 2 [Mupirocin]	*Listeria innocua*	16s rRNA	*Enterococcus*	MALDI-TOF	Enterocin Q	n/a
LW003	mMRS × 2 [Mupirocin]	*Lactobacillus bulgaricus*	16s rRNA	*Enterococcus*	MALDI-TOF	Enterocin 62-6	n/a
DPC7281	WCA	*Fusobacterium nucleatum*	WGS	*Enterococcus* *faecalis*	WGS + BAGEL4	Enterolysin A	*dfrE*, *efrA*, *tetM*, *ermB*, *E. faecalis* chloramphenicol acetyltransferase, *aad*(6), SAT-4, APH(3′)-IIIa
DPC7280	WCA	*Fusobacterium nucleatum*	WGS	*Enterococcus* *faecalis*	WGS + BAGEL4	Enterolysin A Enterocin NKR-5-3B Sactipeptide	*dfrE*, *efrA*
DPC7282	WCA	*Clostridioides difficile*	WGS	*Enterococcus* *mundtii*	WGS + BAGEL4	Enterocin CRL35	No AMR genes identified

**Table 4:** The total amount of BLAST that aligned to the *Enterococcus* spp. pangenomes, recovered from targeted binning.

Species	Total Number of BLAST Hits
*Enterococcus mundtii*	2,732,299
*Enterococcus hirae*	13,166,169
*Enterococcus durans*	9,623,516
*Enterococcus faecalis*	73,372,776
*Enterococcus faecium*	670,879,682

## Data Availability

Raw reads and assembled contigs are available under the accession number PRJEB48530.

## Supplementary Materials

The following are available online at https://www.mdpi.com/article/10.3390/microorganisms10040701/s1, Excel S1. MAG bins recovered with Targeted Approach, Figure S1. MALDI-TOF MS analysis of potential bacteriocin producing gut isolates, Figure S2. Bar chart representing the BLAST hits per sample recovered, for each species of interest, Figure S3. Multidimensional scalling analysis of Bray Curtis distance, at species level between elite Irish athletes and Low BMI non-athlete controls.
