# Supplementary material for: In Vitro and In Silico Based Approaches to Identify Potential Novel Bacteriocins from the Athlete Gut Microbiome of an Elite Athlete Cohort
Source: Microorganisms. 2022 Mar 24;10(4):701. doi: 10.3390/microorganisms10040701 (PMC9025905; doi:10.3390/microorganisms10040701)
Supplement: Supplementary file 1 [file microorganisms-10-00701-s001.zip › Supplementary_Figures.pdf]

Supplementary Material:

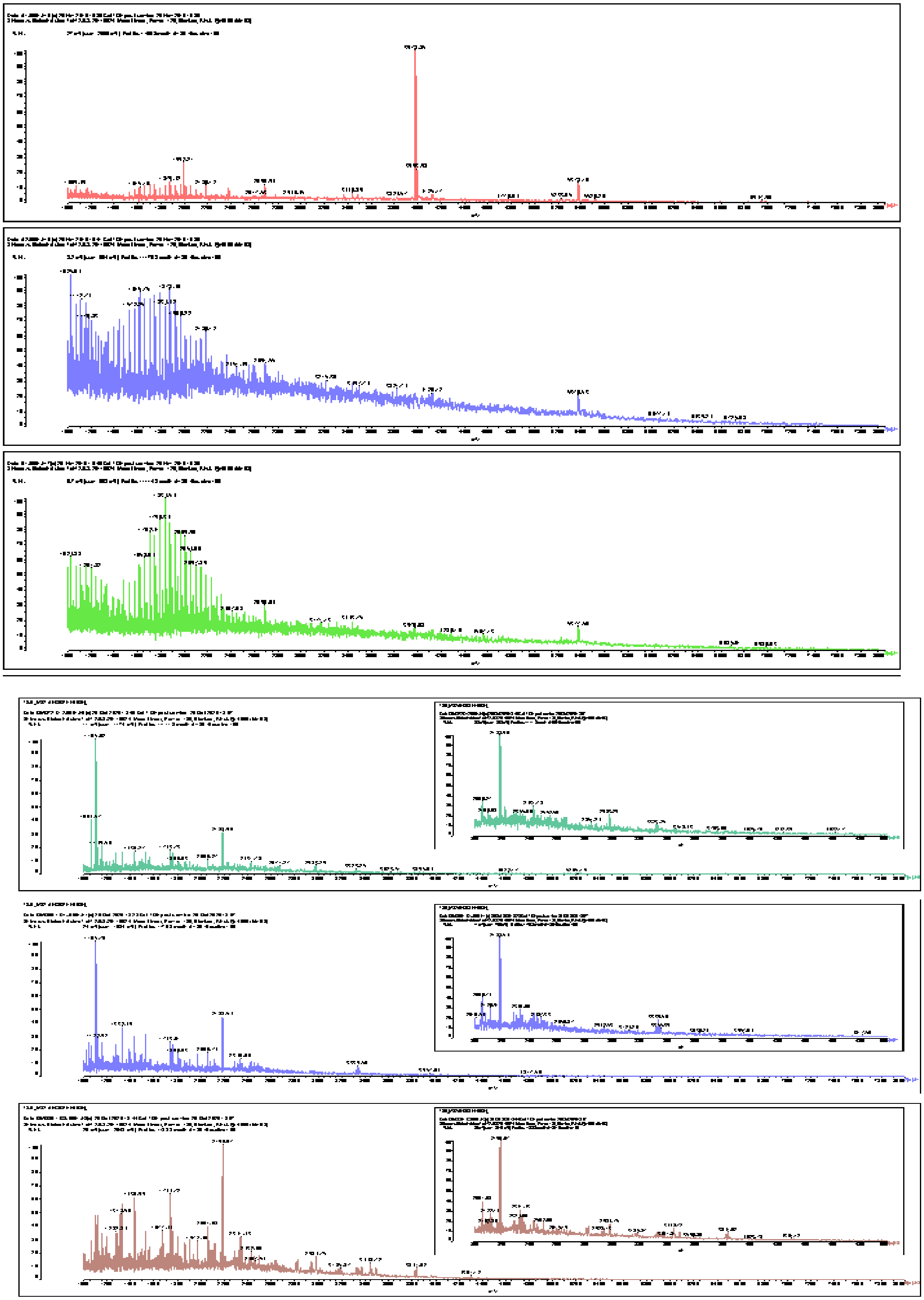

Figure S1. MALDI-TOF MS analysis of potential bacteriocin-producing gut isolates.

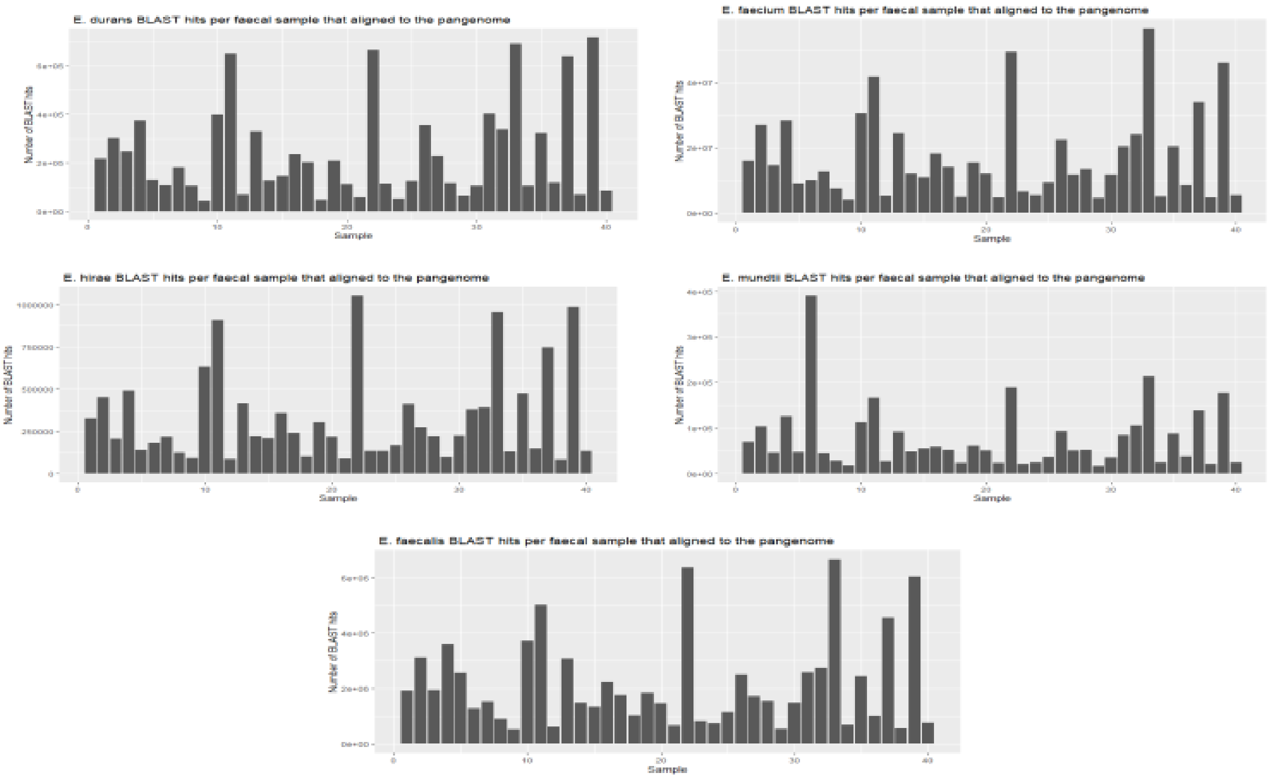

Figure S2. Bar chart representing the BLAST hits per sample recovered, for each species of interest**.**

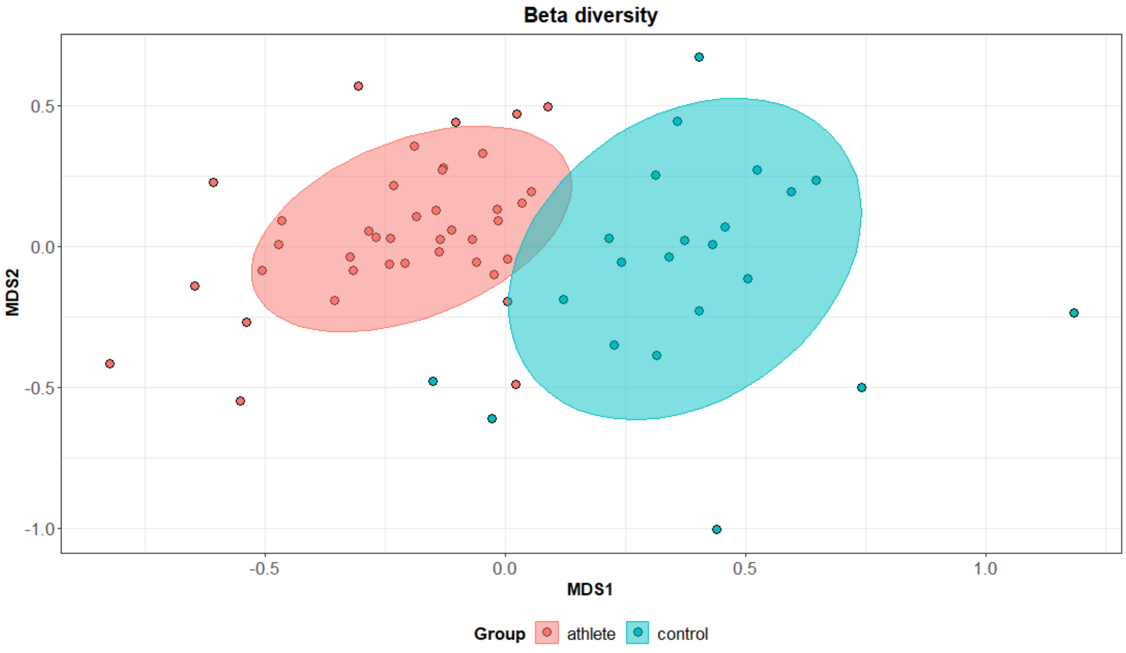

Figure S3. Multidimensional Scaling analysis of Bray-Curtis distance, at species level, between elite Irish athletes and Low BMI non-athlete controls
